# Broader health coverage is good for the nation's health: evidence from country level panel data

**DOI:** 10.1111/rssa.12048

**Published:** 2014-02-19

**Authors:** Rodrigo Moreno-Serra, Peter C Smith

**Affiliations:** Imperial College LondonUK

**Keywords:** Health coverage, Health financing, Mortality, Panel data econometrics, Reverse causality

## Abstract

Progress towards universal health coverage involves providing people with access to needed health services without entailing financial hardship and is often advocated on the grounds that it improves population health. The paper offers econometric evidence on the effects of health coverage on mortality outcomes at the national level. We use a large panel data set of countries, examined by using instrumental variable specifications that explicitly allow for potential reverse causality and unobserved country-specific characteristics. We employ various proxies for the coverage level in a health system. Our results indicate that expanded health coverage, particularly through higher levels of publicly funded health spending, results in lower child and adult mortality, with the beneficial effect on child mortality being larger in poorer countries.

## Introduction

The potential social and economic consequences of broader access to health services have been highlighted by several commentators. For example, an influential report concluded that

‘ … extending the coverage of crucial health services … to the world's poor could save millions of lives each year, reduce poverty, spur economic development, and promote global security’

(Commission on Macroeconomics and Health (2001), foreword). The growing interest in the performance of countries in ensuring adequate access to health services has led to the development of the concept of ‘health system coverage’, the extent to which citizens can secure effective access to healthcare and enjoy protection from exposure to the financial risk that is associated with ill health (Shengelia *et al*., 2003; World Health Organization, 2010). It is often argued that, although most of a country's population can potentially secure health and economic benefits from improved coverage, the gains are likely to be even larger for the poorer and less healthy individuals ( World Health Organization, [Bibr b37],[Bibr b38]). Relative to their richer and healthier counterparts, these groups are, in general, at higher risk of being deterred from seeking timely medical care and less able to secure access to insurance and credit mechanisms that manage uncertainty about healthcare needs and costs (Preker *et al*., 2003).

Consequently, there have been repeated international calls for countries to move towards achieving ‘universal health coverage’, which is commonly defined as providing all people with access to needed health services of sufficient quality to be effective, without entailing financial hardship (World Health Organization, 2010). Higher access to basic health services and higher levels and shares of pooled prepaid health financing (i.e. funds paid by citizens before the need for medical care) are regarded as key dimensions of broader health coverage. The concept of universal health coverage received heightened attention when the World Health Organization 64th World Health Assembly recommended that the topic should be further discussed at the United Nations General Assembly, in light of efforts to achieve the millennium development goals (World Health Organization, 2011a).

A crucial premise underlying the push for universal health coverage is that expansions in health system coverage will necessarily lead to improvements in population health outcomes (see, for example, World Health Organization (2008, 2010) and Garrett *et al*. (2009)). Health coverage as defined above is a multi-dimensional construct, embracing both access to services and financial protection, and there is no single agreed measure of the level of coverage within a country. It is commonly measured by a range of partial indicators. These include the extent of pooled prepaid health spending, the prevalence of out-of-pocket (OOP) and ‘catastrophic’ spending (defined as health spending crossing a prespecified threshold share of a household's disposable income) and utilization rates of key health services. Although the link between system coverage and population health has played a crucial role in the policy debate, the expected relationship between outcomes and coverage indicators is in fact ambiguous *a priori*. For example, an increase in government health expenditure (which normally takes the form of pooled prepaid funds and makes up the majority of national health spending) may in theory be accompanied by a matching reduction in prepaid private health expenditures, which could in principle result in no changes in total pooled spending, and no significant changes in healthcare consumption and health status. Even if government spending increases the total pool of resources devoted to healthcare, the consequences in terms of population health may be disappointing if the additional funds are spent inefficiently, or on population groups that already enjoy good health.

Some of the available empirical literature reflects these ambiguous predictions, as we explain in Moreno-Serra and Smith (2012). Early research in the area focused on identifying simple correlations between pooled health expenditures—usually publicly funded—and outcomes in cross-country data, finding no systematic evidence of an effect on mortality indicators such as child death rates (Musgrove, 1996). The same is true of a more rigorous empirical study by Filmer and Pritchett (1999), which found at best very small public spending impacts on under-5-years (‘under-5’) and infant mortality. More recently, though, a few econometric studies using panel data have found evidence of higher publicly pooled spending leading to better mortality outcomes. Wagstaff and Claeson (2004), examining data for up to 120 countries, estimated reductions ranging between 0.8% and 1.5% in under-5 mortality for a 10% higher share of government health expenditure relative to gross domestic product (GDP). Bokhari *et al*. (2007) used instrumental variable (IV) regressions on a sample of 127 countries to estimate that a 10% increase in *per capita* government health expenditure leads to average reductions of 3.3% and 5% in under-5 and maternal mortality rates respectively. It has also been found that the positive health effects from increased pooled spending tend to vary across population groups and countries, with poor people in poorer countries benefiting the most from additional spending (Bidani and Ravallion, 1997).

There is a growing econometric literature examining the relationship between health outcomes and pooled prepayment in the form of health insurance arrangements. This literature (mostly country case-studies) generally finds improvements in healthcare use and health status due to insurance coverage. This is so, for example, in the USA, where various studies (reviewed in Gruber (2009)) have linked better adult and infant health outcomes to the implementation and expansion of the Medicare and Medicaid schemes. Similarly, the introduction of the universal coverage scheme in Thailand has resulted in an estimated decrease of 6.5 infant deaths per 1000 births among the poor from 2001 to 2005 (Gruber *et al*., 2012). Nonetheless, various other studies have failed to find systematic evidence of causal links between expanded health insurance mechanisms and outcomes (see for example Escobar *et al*. (2010)).

Establishing the direction and strength of the effect of health coverage on population outcomes is essentially an empirical matter. This is not, however, a trivial task, and most of the studies that were mentioned above exhibit important methodological limitations. The main concern is the potential endogeneity of the indicators that are used to measure health coverage. It is possible, for instance, that countries with unobserved factors causing poor health outcomes might decide to broaden health system coverage through higher pooled spending to deal with the problem, potentially leading to a spurious correlation in the data between higher pooled spending and worse population health, and hence masking any beneficial effects of additional expenditures on outcomes. This possibility has long been recognized in the empirical literature, yet most studies to date have been unable to address such simultaneity issues explicitly, and their empirical conclusions may consequently be subject to substantial biases. In this scenario, simple ordinary least squares regressions will merely reflect correlations in the data. Even when more sophisticated methods have been used (such as IV estimators), the analyses have normally relied on cross-sectional data or focused on specific countries or insurance interventions. These factors make it difficult to rule out the possibility of reverse causality and unobserved factors, or to generalize empirical findings to other settings.

The main contribution of this study is to offer robust econometric evidence on the direction and magnitude of health system coverage effects on mortality outcomes. We do so by using a large panel data set of countries with annual data for a period of 14 years (1995–2008), analysed through IV specifications that allow for potential reverse causality and unobserved country-specific characteristics. The two-step IV strategy that is adopted starts by directly and consistently estimating any reverse causal effects of mortality on system coverage measures. Thus, as a further contribution to the field, the empirical approach can shed light on the existence and magnitude of simultaneity between population health and system coverage, which can then be explicitly accounted for in the second estimation step that assesses the effects of coverage on population outcomes. We employ various pooled health financing and access indicators as proxies for the level of coverage in a health system, reflecting its two main dimensions of effective access to needed care and protection from health-related financial hardship. We find that expansions in health system coverage, particularly through higher publicly pooled health spending, result in lower child and adult mortality. We also find that the beneficial effect of pooled health funds on child mortality is only unearthed once the important reverse causal effect of mortality on pooled expenditures has been taken into account, and that the spending impact is larger in poorer countries.

The paper is organized as follows. Section Methodology outlines our econometric methodology. Section Data describes the data set that is used in the analyses. Section Results presents the results of our main estimations and some specification and robustness checks. Section Discussion and conclusions presents a discussion of our empirical results and concludes.

The data that are analysed in the paper and the programs that were used to analyse them can be obtained from

http://wileyonlinelibrary.com/journal/rss-datasets

## Methodology

Our main objective is to obtain credible estimates of the effects of health coverage indicators on population outcomes. However, this is difficult by using a cross-sectional econometric analysis at the country level, primarily because of the possibility of reverse causality or simultaneity in the relationship between coverage and outcomes. A related concern is that there may also be unobservable variables that are correlated both with the level of coverage in a country and the outcomes of interest. The potential endogeneity of coverage indicators would lead to biased estimated coefficients if not taken into account in the regressions.

One way to address the problem of omitted variables is to follow much of the previous literature and to take advantage of longitudinal country level data to look at *changes* over time in health coverage indicators, to eliminate the effect of time invariant omitted variables. However, coverage measures will still be endogenous in the presence of reverse causality between coverage and outcomes, or time varying confounders, or measurement error in the reporting of coverage indicators for particular countries. In what follows, we describe the various strategies that we use to deal with these issues.

### Standard fixed effects models and reverse causality

Consider a simple model where the population health outcome of interest in country *i* at time *t*,* y*_*it*_, depends on a vector *C*_*it*_ containing indicators of the level of health system coverage, and a vector *X*_*it*_ of covariates that might potentially influence both the outcome and health coverage. As detailed in the next section, we measure population health through mortality rates, and coverage through health spending measures and immunization rates. This simple model can be written as

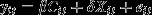
1where *e*_*it*_ is an error term capturing unobservable variables and random noise. We can decompose the error term in equation (1) into three components that account for different sources of endogeneity. Let the error term be denoted as

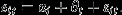
2In this formulation, *α*_*i*_ is a country-specific effect that captures time invariant unobservables that are potentially correlated with the levels of coverage and mortality observed in a given country. The term *θ*_*t*_ is a period-specific intercept that captures aggregate shocks affecting mortality outcomes in all countries at the same time. If all potential sources of endogeneity have been accounted for in the previous two components, *ɛ*_*it*_ represents a random, idiosyncratic error component. This formulation of the error term leads, by substituting equation (2) into equation (1), to the model

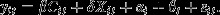
3This model can be estimated through a fixed effects approach. In this case, the within-country variation in outcomes and regressors over time is used to obtain the coefficients of interest. Since the country-specific effect *α*_*i*_ is fixed over time, the effects of time invariant unobservables captured by *α*_*i*_ are eliminated in the estimation. Fixed effects estimations with longitudinal data require the estimated standard errors to be adjusted for arbitrary types of serial correlation and heteroscedasticity (Bertrand *et al*., 2004), so in all econometric analyses in this paper we use panel robust standard errors clustered at the country level.

The main concern is that our variables of interest, the coverage indicators contained in *C*_*it*_, may be correlated with the error term even after removing unobserved time invariant country factors, owing to reverse causality or simultaneity. In this case, estimating equation (3) with fixed effects by pooled ordinary least squares would lead to biased estimates of the *β*-coefficients.

### Dealing with reverse causality: an instrumental variables approach

We adopt a two-step IV approach that seeks firstly to estimate any reverse causal effects of mortality outcomes on coverage indicators, to adjust for these effects directly when estimating the effects of health coverage on mortality in a second step. This methodology to deal with the simultaneous determination of dependent variable and regressors has been suggested and applied in a different context by Brückner (2011) but has not, to the best of our knowledge, been used to analyse the research questions in our paper. Applied to our data and research context, the IV strategy that is adopted makes it possible to investigate further and to tackle explicitly the influence of endogeneity arising from reverse causality.

#### Step 1: estimation of the (reverse) causal effect of mortality outcomes on health coverage

Consider now a potential reverse causality of (say) poor mortality outcomes leading to increased coverage. Let this relationship be expressed as

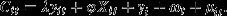
4As before, although country-specific and time-specific effects are included in the model, endogeneity may still be present when health coverage is examined as a function of mortality. So we use an IV strategy that addresses these endogeneity concerns to estimate the effects of our mortality outcomes on health coverage indicators consistently.

An unbiased and consistent IV estimator requires that we identify one or more variables *Z*_*it*_—the instruments—that are sources of exogenous variation in the mortality outcome *y*_*it*_. Specifically, the instruments must be

- *external* to mortality and coverage, i.e. they must not be affected by the mortality and coverage measures, and
- *orthogonal* to coverage, i.e. they must have an effect on coverage indicators only through their effect on the mortality outcome (and not have by themselves a direct effect on coverage measures).

If the identified instruments are valid according to the previous criteria, and relevant in the sense of being reasonably correlated with the instrumented mortality indicator, they should allow us to obtain unbiased regression coefficients from an IV estimation of equation (4). This can be done through two-stage least squares (which we call method IV-2SLS) in the case of a single instrument, or by using the more efficient two-step generalized method-of-moments (which we call method IV-GMM) estimator in the case of more than one IV (Cameron and Trivedi, 2005; Stock and Watson, 2000).

We use two variables as instruments for the country's mortality level in a given year. The first is annual carbon dioxide (CO_2_) emissions *per capita*, which can in principle be correlated with mortality outcomes in opposite ways. Higher outdoor air pollution is often linked to a higher incidence of potentially fatal conditions such as lower respiratory infections and chronic obstructive pulmonary disease, in both infants and adults (Prüss-Üstün and Corvalán, 2006). But higher CO_2_ emissions may also serve as a proxy for larger urban agglomerations. Compared with their rural counterparts, urban populations tend to be less exposed to water-borne conditions and diseases related to vector density (such as malaria, dengue fever, cholera and soil-transmitted helminths), even after differences in education and income have been taken into account (Jamison *et al*., 2006; Aagard-Hansen and Chaignat, 2010). This may lead to a negative association between CO_2_ emissions and child and adult mortality risks. The second instrument that we use is the annual number of battle-related deaths in internal or international conflicts for each country. Conflict deaths typically represent a small fraction of national mortality in our sample (see Section Data) but will tend to be more correlated with mortality in those regions where civil and international wars have been more frequent during our period of analysis, notably sub-Saharan Africa and the Middle East.

The assumption that we make is that differences in both CO_2_ emissions and conflict deaths will induce variations in population health status and mortality rates across countries and within countries over time, which will in turn—and only through their effect on health status—trigger a response in terms of coverage indicators such as pooled health expenditure and rates of immunization. The channels for this response are reasonably intuitive. For instance, higher incidence of respiratory diseases, and associated mortality due to worsening outdoor pollution levels, may induce a governmental response of increased funds directed to the health sector to improve the availability and quality of care for such conditions. In contrast, the expansion of urban agglomerations in a given country over time, proxied by our CO_2_ emissions measure, may contribute to reductions in the morbidity and mortality burdens that are associated with water-borne and vector-transmitted diseases, allowing governments to divert resources away from the health system (e.g. towards housing infrastructure). Finally, countries that have recently been involved in an armed conflict are likely to receive increased health sector support from international donors (and increase spending from domestic sources when feasible) as a response to the deterioration of population health, thus resulting in a rise in pooled health expenditure and public health initiatives like immunization campaigns.

It seems reasonable to think that system coverage responses to variations in CO_2_ emissions and battle-related deaths will be caused primarily by the health effects of the indicators for CO_2_ emissions and battle-related deaths. In particular, although CO_2_ emissions may be related to a country's GDP (which in turn may affect coverage indicators such as government health spending; see Acemoglu *et al*. (2013) and Brückner *et al*. (2012)), all our regressions control for GDP *per capita*, so that any indirect coverage effect of CO_2_ emissions through GDP is taken into account and should not invalidate our instrument. In the same vein, the inclusion of GDP *per capita* in all regressions should account for any indirect effect (through changes in national income) of civil and international conflicts on coverage indicators. Moreover, for our instruments to work less hard on overcoming unobserved heterogeneity, we have included other covariates to capture the influence of potentially important time varying and time invariant country factors (see below), so the instruments need only to be valid conditionally on these covariates. Finally, we also present regression-based evidence supporting the exogeneity and relevance of the two instruments that we use. Their relevance is assessed through *F*-tests of their joint significance in the IV regressions, and through a *χ*^2^ underidentification test that was proposed by Angrist and Pischke (2009) which accounts for the clustered structure of the error terms in the estimated equations. A cluster robust version of Hansen's overidentification *J*-test is used to check the exogeneity of the instruments in the estimated models formally (Cameron and Trivedi, 2005).

For each pairwise combination of mortality and coverage indicators, we estimate a separate version of equation (4) using method IV-GMM to obtain consistent estimates of the reverse causal effect of mortality on coverage, *λ*. By inspecting the sign and statistical significance of *λ*, we can infer the direction of the bias that would affect the coefficients of our coverage variables if we used a standard least squares fixed effects estimator on equation (3). A positive *λ* would mean that higher mortality causally leads to higher coverage, thus resulting in an estimated coverage coefficient *β* in equation (3) that is biased upwards, i.e. closer to 0 or ‘more positive’ than its true value. Conversely, a negative *λ* would imply a negative causal effect of mortality on coverage, leading to a standard fixed effects estimate of the effect of coverage in equation (3) that is biased downwards.

#### Step 2: estimation of the causal effect of health coverage on mortality outcomes

Our strategy is to use the results from the consistent estimation of equation (4) to avoid the potential simultaneity and other biases that were described above. This is done, in a second step, by estimating equation (3) through an IV procedure that expunges the reverse effect of mortality on coverage measures. We use the IV mortality coefficients *λ* estimated from equation (4) to construct adjusted series of coverage indicators 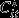 for each country, subtracting the effect of mortality on each coverage indicator:

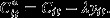
5We then use 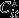 as an instrument for the corresponding coverage indicator in equation (3). Since this procedure leads to only one constructed instrument for each coverage measure, equation (3) is estimated through the IV-2SLS approach. We formally examine the relevance of the generated instruments through the Lagrange multiplier version of the underidentification test that was proposed by Kleibergen and Paap (2006), which is appropriate for the case of two or more instrumented regressors in each equation.

Our IV estimator will be free from any reverse causality bias by construction. Moreover, this two-step IV procedure should in principle be better equipped than the standard fixed effects estimator to account for other potential sources of endogeneity in our context. Even if bias due to reverse causality is not present in some models, the IV estimator will still offer consistent—if less efficient—estimates of health coverage effects, provided that the instruments are valid and the error terms in equations (3) and (4) are uncorrelated, i.e. that there remain no omitted variables affecting both changes in mortality and changes in coverage. If the error terms in equations (3) and (4) remained correlated even after IV estimation, our estimates would be free from simultaneity bias but still be subject to omitted variable bias, the magnitude of which is uncertain *a priori* (Brückner, 2011).

Since the absence of correlation between the two relevant error terms represents an assumption, it cannot be directly tested. We nevertheless undertake extensive diagnostic checks and proxy testing to ascertain the validity of the IV approach that is adopted. Furthermore, it should be emphasized that correlation between the error terms in the two estimated equations will exist only if any remaining omitted variables affect within-country changes in both coverage indicators and mortality *simultaneously*. Omitted variables that affect only one or the other will not cause bias. To make it more likely that the assumption of uncorrelated error terms in equations (3) and (4) holds, we include in the IV regressions some time varying controls that should capture changes in variables that are potentially correlated with both mortality outcomes and health system coverage, such as within-country variations in socio-economic and demographic factors. The introduction of country-specific (fixed) effects should also largely pick up unobserved heterogeneity due to national traits that are relatively persistent over time, such as the quality of government and institutions.

## Data

### Period of analysis, definition of variables and sources

We use annual data that are publicly available at the country level from three databases: the World Bank's *world development indicators* (World Bank, 2011a), the World Health Organization's ‘*Global health observatory*’ (World Health Organization, 2011b), and the Institute of Health Metrics and Evaluation's ‘*Global health data exchange*’ (Institute of Health Metrics and Evaluation, 2011). The definitions and sources of all the variables that are used in our empirical study are given in Table A1 in the on-line appendix 1. The period of analysis is 1995–2008, although for adult mortality rates the information for many countries is available for a slightly more restricted period (from 1998–1999 onwards). In total, the data set includes data for 153 countries. Table1 presents descriptive statistics of all the variables, for both the full sample and the subsample of country–year observations with purchasing-power-adjusted GDP *per capita* up to $12195 (the World Bank's gross national income threshold below which countries are included in the low and middle income country (LMIC) group; see World Bank (2011b)).

**Table 1:** Descriptive statistics†

	Results for full sample					Results for LMICs				
	Mean	Standard deviation	Minimum	Maximum	Countries	Mean	Standard deviation	Minimum	Maximum	Countries
*Outcomes*										
Under-5 mortality rate (per 1000)	45.7	48.9	2.9	250.1	153	63.3	49.6	7.0	250.1	116
Female mortality rate (adult, per 1000)	155.8	114.9	39.2	630.5	151	196.1	116.6	54.8	630.5	113
Male mortality rate (adult, per 1000)	226.4	116.9	66.9	628.5	151	271.7	109.8	104.6	628.5	113
*Regressors*										
Government health spending *per capita* ($100)	5.99	8.43	0.00	48.03	153	1.36	1.35	0.00	7.35	116
VHI health spending *per capita* ($100)	0.58	2.10	0.00	23.72	153	0.14	0.40	0.00	3.22	116
OOP health spending *per capita* ($100)	1.88	2.04	0.03	12.63	153	0.85	0.77	0.03	5.57	116
OOP health spending (share of total, 10s of percentage points)	3.4	1.8	0.3	9.4	153	4.0	1.8	0.3	9.4	116
Total health spending *per capita* ($100)	8.71	11.43	0.09	69.22	153	2.45	2.15	0.09	12.06	116
Immunization coverage (10s of percentage points)	8.6	1.4	2.1	9.9	153	8.3	1.6	2.1	9.9	116
GDP *per capita* ($100)	121.15	131.67	2.80	744.22	153	42.37	32.18	2.80	121.37	116
Primary education enrolment rate (10s of percentage points)	8.6	1.6	2.3	10.0	153	8.2	1.7	2.3	10.0	116
Population 0–14 years (10s of percentage points)	3.1	1.0	1.3	5.0	153	3.6	0.8	1.3	5.0	116
Population >65 years (10s of percentage points)	0.8	0.5	0.1	2.1	153	0.5	0.3	0.2	1.7	116
CO_2_ emissions *per capita* (tonnes)	5.2	6.5	0.0	56.3	153	2.0	2.4	0.0	17.1	116
Conflict deaths (per 100000 population)	1.4	25.8	0.0	683.6	153	2.1	31.3	0.0	683.6	116

† The time period is 1995–2008. The table presents the mean, standard deviation, minimum and maximum values, and number of countries, for the corresponding variable in the full sample and separately for the subsample of LMICs (GDP *per capita* up to $12195).

### Mortality outcomes

We measure population health status through three annual mortality indicators: under-5 mortality rates (deaths per 1000 live births), and female and male adult mortality rates (deaths per 1000). The indicators that are available can be regarded as measures of the overall performance of a health system, after controlling for other factors such as socio-economic and demographic characteristics. Under-5 mortality tends to react relatively quickly to improvements in access to and quality of healthcare provision and was selected by the international community as a key outcome within the millennium development goals framework (United Nations, 2011; World Health Organization, 2008). Unsurprisingly, mortality indicators tend to be worse in the LMIC group compared with the averages in the full sample that includes high income nations.

### Health system coverage indicators

To the extent that existing data permit, we seek to capture the different dimensions of health coverage (as defined in Section Introduction) through measures of the level and proportion of pooled prepaid funds in health financing, and the actual provision of health services. The first indicator is government health expenditure *per capita*. Public spending represents the majority of health resources in most countries and is predominantly made up of pooled funds prepaid by citizens through channels such as social health insurance contributions or taxes. It also includes donor funds channelled through government spending. Incremental publicly pooled spending often takes the form of the introduction of national health insurance schemes or their expansion to previously uncovered groups. The available evidence indicates important improvements in access to care and financial risk protection arising from increased pooled health financing and broader public insurance arrangements (Levy and Meltzer, 2004; Gruber, 2009; Escobar *et al*., 2010).

We also include the two components of private health spending as separate coverage indicators in the main models. The first component represents privately pooled resources: voluntary health insurance (VHI) spending *per capita*. The second component is non-pooled OOP payments *per capita*. Extra private spending may in principle mean higher consumption of necessary health services regardless of the source of funds (VHI or OOP), so the two indicators serve as further coverage proxies in our baseline specification. Nonetheless, OOP health spending has been shown to be positively correlated with poorer financial protection (and hence poorer coverage), as measured by the incidence of catastrophic health expenditure across countries (Xu *et al*., 2007). The degree of financial protection in a health system is commonly associated with the relative participation of any type of pooled prepaid funds in total health financing ( World Health Organization, 2010a 2012; Xu *et al*., 2007). Thus, to examine the financial protection dimension more directly, we use OOP health spending as a share of total health expenditure (instead of the level of OOP expenditure) in an alternative regression specification.

We aim to capture further the health effect of effective access to care through a constructed immunization indicator. Immunization rates are widely used as markers of the overall performance of health systems in guaranteeing effective access to necessary care (World Health Organization, 2008). In our data set, annual cross-country information for the period of study is available for six immunization rates: diphtheria–tetanus–pertussis, hepatitis type B, *haemophilus influenzae* type B, polio, Bacillus Calmette–Guérin and measles. We summarize the information that is provided by these six indicators by constructing an aggregate immunization rate variable, representing the median rate across the six categories of immunization for a given country–year observation.

Government health spending *per capita* in the whole sample is around three times larger than the average OOP spending *per capita*. OOP spending represents over a third of national health financing on average in the full sample, whereas VHI spending is not a large component of health financing in the vast majority of countries. LMICs exhibit lower rates of immunization and substantially lower *per capita* health spending figures compared with the corresponding full sample averages, with a higher share of OOP payments in total health spending.

### Instruments and remaining covariates

As discussed in Section Dealing with reverse causality: an instrumental variables approach, we use two variables as instruments for the observed mortality rates in each country. The first is annual CO_2_ emissions *per capita* in tonnes. The average of the full sample is more than double that of the LMIC group. The second instrument, annual battle-related deaths in conflicts, exhibits a higher average in the LMIC subsample than in the full sample. These sample averages are still relatively low, however, and are particularly driven by the occurrence of conflicts in low income nations over the period of study. The covariates that are used in the econometric analyses control for cross-country heterogeneity in terms of national income (GDP *per capita*), formal education (the primary education enrolment rate) and demographic profile (shares of population aged 0–14 and over 65 years). All models estimated in this paper also include a full set of year-specific indicators (time dummy variables) to capture the effects of common aggregate shocks during the study period.

## Results

### Preliminary analyses: what the raw data tell us

We first examine raw correlations between changes in our coverage measures between 1995 and 2008 and changes in outcomes. We use 5-year average values of coverage indicators for the period 1995–1999 as the initial data points, and average values for 2004–2008 as the final data points. We then divide countries into terciles of increase in each coverage indicator, where the bottom tercile comprises those countries with the smallest increases in coverage between the initial and final periods, whereas the top quartile contains those countries with the largest increases in coverage in the same time span.

**Figure 1:**
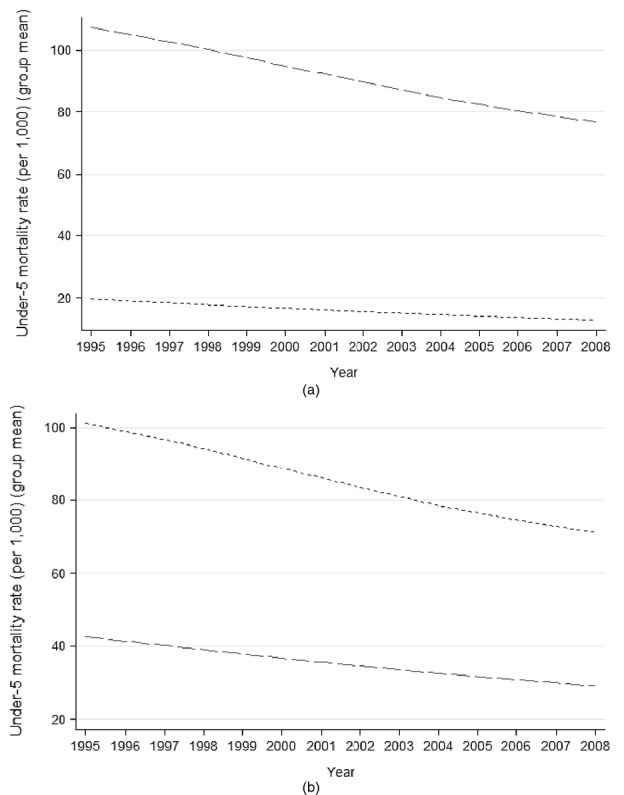
Average under-5 mortality over time across groups (terciles) of countries with the largest and smallest increases in health coverage indicators, 1995–2008 (— —, bottom tercile of increase; ------, top tercile of increase): (a) terciles of increases in mean government expenditure on health ($100 *per capita*), from 1995–1999 to 2004–2008; (b) terciles of increase in mean immunization coverage (10s of percentage points), from 1995–1999 to 2004–2008

For brevity, Fig. 1 shows only the evolution of under-5 mortality rates across the bottom and top tercile groups of increase in government health expenditure (Fig. 1(a)) and immunization coverage (Fig. 1(b)). These unadjusted comparisons show that countries in the bottom tercile of increase in government expenditure *per capita* actually exhibited *faster* decreases in under-5 mortality. Moreover, mortality rates for female and male adults do not seem to have followed different paths according to changes in health spending (the graphs are not shown). In contrast, under-5 mortality—and adult mortality—improved faster over the period in countries with the largest expansions in immunization rates.

Although in the raw data larger increases in government health expenditure *per capita* seem to have little association with improvements in mortality, it is noteworthy that the group of countries with the largest increases in government spending is formed mainly by high income countries. These started the period at a much lower baseline in terms of mortality levels, compared with countries in the bottom tercile of increase in spending. It seems plausible to argue that $1 of extra public spending might yield higher marginal returns in terms of outcomes for lower income countries compared with richer nations, thus helping to explain this result and indicating the importance of adjusting the coverage–outcome relationships for differences in national income, as well as examining the possibility of differential effects of health coverage expansions according to national income levels.

### Main regression results

#### The causal effect of mortality outcomes on health coverage

The first step of our IV approach, which was outlined in Section Dealing with reverse causality: an instrumental variables approach, involves the estimation of the reverse causal effects of mortality outcomes on each of our health spending and immunization variables. For each coverage measure as dependent variable, we estimate three separate versions of equation (4)—each having one of the mortality rates as regressor of interest, plus the other covariates that were described above—using an IV-GMM procedure. In each equation, the corresponding mortality rate is instrumented by our two chosen instruments: CO_2_ emissions and conflict deaths. The IV models perform well in this first step, according to tests for both the relevance and the validity of the instrument set. The results of these tests are presented and discussed in more detail in the on-line appendix 2.

Generally, the response of coverage indicators to changes in mortality rates does not appear to be substantial. The estimated mortality coefficients (the *λ* from equation (4)) normally fail to be statistically significant at conventional levels, as shown in Table A2 (in the on-line appendix 2). However, in one particular instance—the effect of under-5 mortality variations on government health spending—the point estimate is relatively sizable and comes very close to conventional statistical significance. It implies that an increase of 1 standard deviation in under-5 mortality (48.9 deaths per 1000) leads to an average governmental response of around a $0.25 increase in health spending per person, or $8.1 million in total public health expenditure. For comparison, this spending effect is roughly 25 times larger than the measured governmental response to a 1-standard-deviation increase in adult male mortality (around $0.01 *per capita*, or $0.3 million in total).

The low *p*-value and positive sign of the estimated under-5 mortality coefficient, coupled with the generally positive sign of the coefficients of the other two mortality variables in on-line appendix Table A2, suggest the possibility of obtaining standard (non-IV) fixed effects estimates of the effect of coverage measures on mortality that are biased towards 0 or positive values. This adds to the potential biases arising from time varying omitted variables and measurement error, to which the standard fixed effects estimator is particularly vulnerable compared with IV estimation.

#### The causal effect of health coverage on mortality outcomes

We present in Table2 the results from the estimation of equation (3) for each mortality outcome. The first column for a given outcome shows the results of the standard least squares fixed effects estimation, whereas the second column shows the corresponding IV estimation results. The latter are obtained by instrumenting each of the coverage indicators in the regression by their generated, reverse-causality-adjusted counterpart obtained from the first IV step previously described. So, for example, column (2) presents the IV-2SLS-estimated effects of coverage measures on under-5 mortality, where the instrument for government health spending is generated as in equation (5), and likewise for the other spending and immunization variables. To make the interpretation of estimated coefficients and cross-country comparisons more intuitive, we use natural units of the variables and rescale our rate and share regressors to be expressed as tenths (e.g. 10 percentage points of immunization coverage), and *per capita* variables to be expressed as hundreds (e.g. $100 of government health spending *per capita*).

**Table 2:** Baseline results for the effects of health coverage on mortality†

	Results for under-5 mortality rate		Results for female mortality rate (adult)		Results for male mortality rate (adult)	
	FE-LS, (1)	IV-2SLS, (2)	FE-LS, (3)	IV-2SLS, (4)	FE-LS, (5)	IV-2SLS, (6)
Government health	*0.581*	−*13.193*	−1.218	−*2.583*	−*1.019*	−*2.210*
spending *per capita*	(*0.009*)	(*0.018*)	(0.102)	(*0.050*)	(*0.070*)	(*0.025*)
VHI health spending	0.556	−6.143	0.680	5.153	0.595	8.731
	(0.155)	(0.507)	(0.542)	(0.161)	(0.485)	(0.172)
OOP health spending	0.856	2.685	−0.754	−*23.385*	−1.487	−*15.545*
	(0.179)	(0.594)	(0.753)	(*0.040*)	(0.530)	(*0.016*)
Immunization coverage	−*1.962*	−*2.203*	−1.957	−*9.841*	−1.123	−*7.858*
	(*0.000*)	(*0.073*)	(0.242)	(*0.030*)	(0.450)	(*0.020*)
Country fixed effects	Yes	Yes	Yes	Yes	Yes	Yes
Year fixed effects	Yes	Yes	Yes	Yes	Yes	Yes
1st-stage underidentification Lagrange multiplier test(statistic)		8.50		46.81		31.75
1st-stage underidentification Lagrange multiplier test (*p*-value)		0.004		0.000		0.000
*F*-statistic	17.75	3.95	11.52	4.40	23.68	9.89
*F*-statistic (*p*-value)	0.000	0.000	0.000	0.000	0.000	0.000
Number of countries	153	153	148	148	148	148
Observations	1397	1397	1222	1222	1222	1222

† The time period is 1995–2008. The models were estimated by standard least squares fixed effects (method FE-LS) or IVs through a two-stage least squares approach (method IV-2SLS), using as instruments the reverse-causality-adjusted coverage indicators (see the text). All regressions also control for GDP *per capita*, the primary education enrolment rate, the share of population aged 0–14 years and the share of population aged over 65 years. *p*-values (in parentheses under the coefficients) are from two-sided *t*-tests with standard errors robust to arbitrary heteroscedasticity and auto-correlation. Entries in italics indicate coefficients that are statistically significant at the 10% level of confidence or below.

We formally assess the adequacy of IV modelling in our data, compared with standard least squares fixed effects estimation, by assessing the likely exogeneity of coverage indicators through tests of difference in Hansen statistics (Baum *et al*., 2007; Schaffer, 2012). These tests strongly reject the exogeneity of the coverage variables in all three mortality regressions with *p*-values lower than 0.025, thus pointing to the need for IV estimation as opposed to a standard fixed effects strategy. Also, as shown in the bottom section of Table2, in all instances the statistical tests support the relevance of the generated instruments, strongly rejecting underidentification in the first stage of the IV estimations.

The focus here is on the estimated effects of our proxies for the various dimensions of health system coverage: government, VHI and OOP health spending *per capita*, and the rate of immunization. In addition to these variables and country-specific effects, all regressions control for GDP *per capita*, the primary education enrolment rate, the share of population aged 0–14 years, the share of population aged over 65 years and a full set of year dummy variables (the results for these covariates have been omitted for conciseness).

##### Pooled prepaid health spending

The preferred IV estimations strongly indicate that higher levels of government health expenditure *per capita* lead to better population outcomes, measured either by under-5 or adult mortality rates. The point estimates, which are all statistically significant at the 5% level, imply economically important effects: a $100 increase in government spending *per capita* results in a reduction of 13.2 per 1000 in under-5 mortality (Table2, column (2)), as well as decreases of around 2.6 and 2.2 per 1000 in the mortality rates for adult females and males respectively (columns (4) and (6)). Using an IV estimator to account for the putative endogeneity of government health spending leads to larger estimated effects of public spending on mortality, compared with the equivalent standard fixed effects estimates. The most noteworthy case is that of under-5 mortality, for which we found stronger evidence of reverse causality in the first step of the IV modelling. Although the standard fixed effects estimator counterintuitively suggests a positive and statistically significant effect of public health spending on deaths in children under 5 years old, accounting for any reverse causality or omitted variable bias through IV leads to a large and negative point estimate. This confirms the expected upward bias of the standard fixed effects estimate, which is suggested in on-line appendix Table A2. As for privately pooled health expenditure, there is no evidence that variations in VHI spending are related to variations in mortality outcomes. In all instances the estimated IV (or standard fixed effects) coefficients are nowhere near statistical significance.

##### Private out-of-pocket health spending

The IV estimations suggest a negative effect of private OOP health spending on adult mortality. The point estimates are statistically significant at the 5% level and indicate reductions of around 23.4 female and 15.6 male deaths per 1000 in response to a $100 higher *per capita* OOP expenditure (Table2, columns (4) and (6)). These effects are much larger than those implied by the (statistically insignificant) standard fixed effects estimates. They are also substantially larger than the corresponding adult mortality estimates for government health spending. The comparative efficiency of incremental health financing through public as opposed to private sources is a matter that we further explore below. In contrast, higher OOP spending does not seem to affect under-5 mortality systematically, judged by the small and statistically insignificant point estimates.

##### Immunization coverage

As in the case of government spending, the results that are shown in Table2 for the rate of immunization strongly indicate that expansions in health coverage *per se* lead to improvements in population health. The statistically significant IV coefficients suggest reductions of around 2.2 under-5 deaths per 1000 live births (column (2)), and 9.8 and 7.9 fewer female and male adult deaths per 1000 (respectively; columns (4) and (6)), in response to an increase of 10 percentage points in the immunization coverage rate. Once again, IV estimation generates larger point estimates than those from the standard fixed effects model that makes no adjustment for reverse causality, in particular for adult mortality outcomes.

### Further specification and robustness checks

In this section, we undertake a battery of tests to check whether the health coverage effects that were identified previously are robust to changes in the econometric specification and estimation sample. Our focus is on the stability of the statistically significant coefficients of health coverage indicators found in the preferred IV-2SLS estimations (Table2). Therefore, in Table3 we display the results of the specification and robustness checks for three indicators: government health spending, OOP health spending and immunization coverage. For each of these variables, the first row shows the statistically significant baseline coefficients that were found in the IV models from Table2, followed by the corresponding coefficients and statistical significance levels estimated in each specification and robustness test. All models in this section are estimated by using our IV-2SLS procedure with the same covariates as before and pass the diagnostic tests that were discussed in the previous sections (the results are not shown).

#### Allowance for lagged coverage effects

It seems reasonable to expect current expansions in health coverage to have different effects over time depending on the health outcome that is analysed. Moreover, for some outcomes, the cumulative health effect of expanded coverage over the years may be larger than any such effects measured contemporaneously. Focusing only on contemporaneous effects may therefore mask important non-linearities in health effects over time.

**Table 3:** Specification and robustness checks for the baseline effects of health coverage on mortality†

	Results for under-5 mortality rate, IV-2SLS, (1)	Results for female mortality rate (adult), IV-2SLS, (2)	Results for male mortality rate (adult), IV-2SLS, (3)
*Government health spending*			
Baseline	−13.193	−2.583	−2.210
	(0.018)	(0.050)	(0.025)
(a) With first and second lags (sum of all coefficients)	−7.860	−1.377	−1.966
	(0.034)	(0.212)	(0.043)
(b) Excluding outliers	−16.291	−2.877	−2.166
	(0.022)	(0.022)	(0.046)
(c) Excluding years before 1999	−8.341	−2.163	−2.303
	(0.026)	(0.050)	(0.015)
*OOP health spending*			
Baseline		−23.385	−15.545
		(0.040)	(0.016)
(a) With first and second lags (sum of all coefficients)		−25.693	−14.928
		(0.085)	(0.048)
(b) Excluding outliers		−14.022	−12.475
		(0.012)	(0.006)
(c) Excluding years before 1999		−22.731	−15.066
		(0.045)	(0.018)
(d) OOP as a share of total health spending		34.196	38.934
		(0.031)	(0.012)
*Immunization coverage*			
Baseline	−2.203	−9.841	−7.858
	(0.073)	(0.030)	(0.020)
(a) With first and second lags (sum of all coefficients)	−0.807	−13.419	−9.870
	(0.503)	(0.052)	(0.043)
(b) Excluding outliers	−1.807	−6.447	−5.947
	(0.212)	(0.027)	(0.016)
(c) Excluding years before 1999	−1.808	−7.968	−5.993
	(0.054)	(0.043)	(0.037)

† For each health coverage indicator, the first row shows the statistically significant two-stage least squares (method IV-2SLS) coefficients found in the baseline models (presented in Table2), followed by the corresponding coefficients estimated in each specification and robustness test. Test (a) adds the first and second lags of the coverage indicators and the estimates refer to the sum of the contemporaneous and lagged effects. Test (b) excludes 41 outlying observations. Test (c) excludes the period 1995–1998 from the estimations. Test (d) includes as coverage measures only OOP health expenditure as a share of the total (instead of OOP spending *per capita*), total health expenditure and the rate of immunization. All regressions also control for GDP *per capita*, the primary education enrolment rate, the share of population aged 0–14 years, the share of population aged over 65 years and country and year fixed effects. *p*-values (in parentheses under the coefficients) are from two-sided *t*-tests with standard errors robust to arbitrary heteroscedasticity and auto-correlation.

To deal with these issues, we estimate a finite distributed lag specification as an extension of our baseline model. Assuming up to 2-year lagged coverage effects, equation (3) can be rewritten as

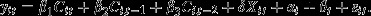
6So we add to our baseline model the first and second lags of each coverage indicator. The cumulative effect of coverage on the health outcome *y*_*it*_ will then be given by the sum of the contemporaneous and lagged estimated effects. We estimate equation (6) through the preferred IV approach, using the first and second lags of the generated variables 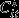 as instruments for the observed lagged coverage values. For each mortality outcome and health coverage variable in Table3, row (a) shows the estimated sum of the coefficients of contemporaneous and lagged effects from equation (6). We have also estimated alternative models by using further lags for each regressor of interest (*t*−3, *t*−4 and *t*−5). In no case did we find statistically significant effects of lagged variables corresponding to years beyond *t*−2 (the results are not shown).

The key message from these estimations is that the baseline mortality results are mostly robust to the inclusion of lagged coverage measures, and the effects of coverage indicators on mortality seem in the main to be adequately captured by the baseline model with contemporaneous effects. There appear to be delayed effects of variations in immunization coverage on adult mortality rates, suggesting that the baseline model may yield underestimated immunization effects for these outcomes. In contrast, the estimated effect of immunization on the under-5 mortality rate, and that of government health spending on adult female mortality, becomes smaller and statistically insignificant at conventional levels (compared with the baseline) when lags are included.

#### Exclusion of outliers

A visual examination of the raw data shows some data points that, given their noteworthy discrepancy from the values that are observed in the rest of the sample, might be distorting the conclusions of our empirical exercise. We use a formal statistical procedure that was proposed by Billor *et al*. (2000) to identify the outliers. The procedure leads to the nomination of 11 country–year observations as mortality outliers, mainly due to adult mortality rates in excess of 500 per 1000: Botswana (years 2001–2006), Swaziland (2004–2007) and South Africa (2007). Another 30 observations for high income countries are considered outliers in terms of our spending indicators: Luxembourg (2002–2006), the Netherlands (2003–2005), Switzerland (1995–1996 and 1999–2007) and the USA (1995–1996 and 1998–2006).

We then rerun our baseline IV-2SLS models excluding the 41 outlying observations. The estimates in Table3 (row (b) for each coverage variable) show that our main IV results for government health expenditure are largely robust to the exclusion of outliers. The new results for OOP spending effects on adult mortality are still statistically significant as well, although the point estimates are somewhat smaller than the corresponding baseline estimates. This suggests that some observations with very high OOP spending values and very low mortality rates are partly driving the baseline estimation results for this particular indicator. The exclusion of outliers makes the baseline estimated effect of immunization coverage on under-5 mortality again lose conventional statistical significance, although its point estimate is close to the baseline.

#### Exclusion of the period before 1999

Our data are notably sparser for the years before 1999, in particular as far as adult mortality rates are concerned: there are usable estimation data for about 61 countries on average between 1995 and 1998. To avoid concerns about bias in our estimations due to the overrepresentation of countries with better reporting systems in the sample before 1999, we estimate our baseline IV specifications by using a restricted sample containing data only for the period 1999–2008. The estimates in Table3 (row (c) for each coverage indicator) are reassuring in terms of the robustness of our baseline results to the use of a shorter period of analysis, with estimated effects generally similar to the baseline results.

#### Share of out-of-pocket health payments

The baseline estimates point to beneficial effects of higher OOP health spending levels on adult mortality. This is consistent with the hypothesis that higher spending, from whatever source, tends to improve outcomes through consumption of necessary healthcare. It has been argued, however, that the incidence of catastrophic health payments—the most commonly used measure of financial risk protection in a health system—is closely and negatively associated with the proportion of national health spending funded privately through OOP payments (see, for example, World Health Organization (2012)). We thus estimate an alternative econometric specification replacing OOP expenditure *per capita* by OOP payments as a share of total health expenditure. Given that private health spending is virtually equivalent to OOP health spending in many countries in the sample, we estimate specifications including as coverage measures only total health expenditure, OOP health expenditure as a share of the total and the immunization rate (in addition to the income, education, demographic and time dummy controls). We generate instruments for these spending measures and perform IV-2SLS estimations as explained in Section Dealing with reverse causality: an instrumental variables approach.

The estimation results for this specification test are presented in Table3 (row (d) for the OOP health spending variable). Keeping the level of national health spending constant, a higher share of OOP spending has a deleterious effect on both adult mortality outcomes. The IV point estimates, which are statistically significant at the 5% level, suggest that an increase of 10 percentage points in the share of OOP health financing leads to increases of over 34 deaths of adult females and males per 1000. To put this result into context, a 10-percentage-point increase in the share of OOP payments over total health expenditure is close to moving from the sample average (share of OOP, 34%) to the average among low income countries (share of OOP, 46%). Therefore, there is strong evidence that health coverage expansions in the form of improved financial risk protection—proxied by reductions in the share of OOP payments in health financing—have a beneficial effect on population health.

#### Allowance for differential coverage effects in low and middle income countries

The examination of the raw data in Section Preliminary analyses: what the raw data tell us suggested the possibility of non-linear effects of health coverage expansions according to national income levels, possibly because poorer countries started the study period with worse mortality conditions and remained so throughout. The inclusion of country-specific effects should capture any confounding influence of initial levels of mortality for the estimation of coverage effects. Furthermore, the inclusion of time varying controls coupled with appropriate instrumentation of coverage measures should pick up the influence of unobservable, temporally correlated country factors that may make some nations more likely to experience (say) high mortality over time, or so-called ‘spurious’ state dependence (Heckman and Borjas, 1980). But we further explore whether, for a given expansion in health coverage, countries that are characterized by lower income and hence higher mortality rates tend to obtain larger marginal health gains than their richer counterparts.

We estimate an extension of equation (3), adding interaction terms between an LMIC indicator (equal to 1 if the country–year observation has a GDP *per capita* up to $12195; 0 otherwise) and each of the health spending and immunization variables, i.e. adding one *C*_*it*_×LMIC_*i*_ term for each coverage measure. The results from the interacted specifications, estimated by method IV-2SLS using the full sample of countries, are presented in Table4. For each mortality outcome, the first column presents the results from model (3) expanded by the inclusion of the four interacted coverage terms. Each interaction term is instrumented by the corresponding generated coverage instrument interacted with LMIC_*i*_. The second column for each adult mortality outcome displays the results from a model where the coverage indicators are the share of OOP health spending over total, total health spending *per capita* and the immunization rate (as in the robustness test in Section 4.3.4), plus their interactions with LMIC_*i*_. The estimated sums of the coefficients of main effects and interaction terms give the total estimated health coverage effects for LMICs.

**Table 4:** Estimates of coverage effects in LMICs†

	Results for under-5 mortality rate, IV-2SLS, (1)	Results for female mortality rate (adult), IV-2SLS		Results for male mortality rate (adult), IV-2SLS	
		(2)	(3)	(4)	(5)
	*Sum of coefficients of main effect and interaction terms*				
Government health spending *per capita*	−*90.772*	−18.414		−12.004	
	(*0.069*)	(0.135)		(0.120)	
OOP health spending *per capita*		−*49.014*		−*37.914*	
		(*0.051*)		(*0.020*)	
Immunization coverage	−0.011	−*9.406*		−*7.185*	
	(0.997)	(*0.036*)		(*0.021*)	
OOP health spending (share of total)			*34.439*		15.902
			(*0.036*)		(0.939)
	Yes	Yes	Yes	Yes	Yes
Year fixed effects	Yes	Yes	Yes	Yes	Yes
1st-stage underidentification Lagrange multiplier test (statistic)	4.96	17.58	1.06	5.39	0.01
1st-stage underidentification Lagrange multiplier test (*p*-value)	0.026	0.000	0.303	0.020	0.911
*F*-statistic: 2nd stage	1.98	2.99	2.05	7.84	2.45
*F*-statistic: 2nd stage (*p*-value)	0.007	0.000	0.006	0.000	0.001
Number of countries	153	148	148	148	148
Observations	1397	1222	1222	1222	1222

† For each health coverage indicator, the table shows the two-stage least squares (method IV-2SLS) estimates from models where the baseline specification is expanded through the inclusion of interaction terms between an indicator for LMIC (equal to 1 if the country–year observation has a GDP *per capita* up to $12195; 0 otherwise) and each of the health spending and immunization variables. All regressions also control for GDP *per capita*, the primary education enrolment rate, the share of population aged 0–14 years and the share of population aged over 65 years. *p*-values (in parentheses under coefficients) are from two-sided *t*-tests with standard errors robust to arbitrary heteroscedasticity and auto-correlation. Entries in italics indicate coefficients that are statistically significant at the 10% level of confidence or below.

The first conclusion from Table4 is that the beneficial effect of higher public health spending on under-5 mortality is substantially larger for LMICs. Column (1) shows an estimated reduction of about 90 deaths of children under 5 years old per 1000 for a $100 increase in government health expenditures: an effect that is over six times larger than the average effect estimated for the full sample of countries. In contrast, there is weaker evidence that additional public health spending has larger adult mortality effects in the LMIC group (columns (2) and (4)). And, although additional OOP health spending *per capita* is also linked to larger adult health benefits in the LMIC group, a higher share of OOP payments in total health financing is once again found to result in poorer outcomes. The latter set of results should nevertheless be treated with caution, since the IV models in columns (3) and (5) are the only ones in our paper for which the null hypothesis of first-stage underidentification cannot be statistically rejected. Finally, the estimates for higher immunization coverage in the LMIC group indicate adult mortality reductions that are similar in size to the baseline results for the full sample, with no evidence of effects in the under-5 mortality model.

## Discussion and conclusions

The pursuit of universal health coverage has become an important policy objective in many countries, given added impetus by publication of World Health Organization (2010). An important justification for the push towards universal coverage is the assumption that it will lead to improvements in population health, which is a fundamental goal of any health system. Credible empirical evidence on this link is still scarce, however. The main purpose of this study is to employ a robust statistical methodology to provide evidence on the causal relationship between national levels of health system coverage and mortality outcomes.

We assemble a large panel data set with data for the period 1995–2008 encompassing 153 countries, and we measure the degree of health system coverage by a range of indicators, including measures of pooled prepaid health expenditure and rates of immunization. To answer our research question reliably, we use a two-step IV approach that directly estimates potential reverse causal effects of under-5 and adult mortality levels on coverage indicators. In contrast with the available literature in the area, our approach allows us to estimate the effect of changes in health outcomes on coverage indicators directly and to adjust explicitly for any such effects when estimating the effects of coverage on mortality, as well as to obtain estimates of coverage effects that are consistent in the presence of relevant omitted variables and measurement error. We subject our baseline empirical models to a battery of specification and robustness tests, including examination of the influence of delayed coverage effects and country income levels, outlying observations, changes in the period of analysis and using a specific proxy for the degree of financial risk protection in the health system.

Our results (which are summarized for convenience in Table5) strongly indicate that expansions in health system coverage through higher publicly pooled spending lead, on average, to improved population health measured by lower child and adult mortality rates. The magnitude of the estimated effects varies according to the specific mortality outcome in question. The estimated gains are the largest when under-5 mortality is examined: on average, a 10% increase in government expenditure *per capita* results in approximate reductions of 7.9 deaths of children under 5 years old per 1000, and at least 1.3 deaths per 1000 in adult mortality rates. If we focus on LMICs, the estimated effect of a 10% increase in public spending is to reduce under-5 mortality by over 12 deaths per 1000: a 1.5-times-larger effect than in the full sample. Importantly, we find that the beneficial and sizable effect of government health spending on under-5 mortality is only uncovered once the positive response of public spending to increased mortality is adjusted for, which may explain some ambiguous results from previous empirical literature in the area (e.g. Musgrove (1996) and Filmer and Pritchett (1999)). Our results are consistent with the intuitive notion that governments and international donors may increase health system funding and service coverage when there is a worsening in child mortality, which is a general and often readily available indicator of population health.

**Table 5:** Summary of results for the effects of health coverage on mortality†

	Results for a 10% increase in the following indicators:				
	Government health spending per capita	VHI health spending per capita	OOP health spending per capita	OOP health spending (share of total)	Immunization coverage rate
Under-5 mortality rate	(−) 7.9 per 1000	No effect	No effect	No effect	Negative significant effect not robust
Female mortality rate (adult)	(−) 1.6 per 1000	No effect	(−) 4.4 per 1000	(+) 11.6 per 1000	(−) 8.5 per 1000
Male mortality rate (adult)	(−) 1.3 per 1000	No effect	(−) 2.9 per 1000	(+) 13.6 per 1000	(−) 6.8 per 1000

† The table presents the baseline estimated incremental effect, on each health outcome, for a 10% increase in the corresponding coverage indicator (relative to the observed average in the data). Models were estimated through two-stage least squares (method IV-2SLS). Incremental effects are expressed in deaths per 1000. ‘(+)’ denotes an increase (a positive regression coefficient) and ‘(−)’ denotes a decrease (a negative regression coefficient). ‘No effect’ denotes that no statistically significant effect was found in the baseline model. ‘Significant effect not robust’ denotes that a statistically significant effect was found in the baseline model but not across robustness tests.

The real world relevance of the estimated pooled health spending effects is not trivial, as a simple calculation of saved life-years can illustrate. According to the coefficients from the baseline IV specification for under-5 mortality, and figures for average country population aged 0–4 years, average under-5 mortality rate and life expectancy at age 5 years, the average country would see a total of 451 lives and 30443 years of life saved for an extra $1 of government health spending *per capita* (see the on-line appendix 3 for details of the calculations). For the average LMIC, the point estimates would imply even larger totals of 3707 lives and 240061 life-years saved per country, for an extra $1 of government spending *per capita*. These calculations imply public health spending figures per life saved of around $72000 for the typical country, and only $9400 per life saved for the typical lower income country. This result also implies that the marginal cost of saving a year of life is on average around $1000 in the whole sample of countries, whereas the analogous figure for an LMIC is just $145. These figures compare very favourably with a widely cited benchmark of $100000 used in high income countries as the implicit value of a year of life in perfect health (Cutler and McClellan, 2001).

In addition to looking at pooled health funds channelled through the public sector, the data allow us to disaggregate private health spending into prepaid VHI and non-pooled OOP payments. For the average country, publicly pooled spending seems far more effective in reducing mortality than pooled private VHI funds, although it should be noted that there is a high frequency of zero VHI values in the sample (particularly in lower income countries), and generally very small non-zero figures (more than half of our country–year observations have VHI expenditure *per capita* that is lower than $0.05), thus making it difficult to identify VHI effects from variations at the aggregate level.

Our analysis yields interesting insights into the aggregate health effects of non-pooled private spending. Higher levels of OOP health spending *per capita* are linked to lower adult mortality, though the baseline estimates are substantially reduced by the removal of outliers from the sample. If we take into account the large, negative estimated effect of public spending on under-5 deaths (compared with no effect of OOP spending on the same outcome), the effects of an additional $1 of pooled and non-pooled health spending on overall mortality are similar in magnitude, with a somewhat larger total effect for pooled spending in elasticity terms (see Table5). But we also find that a higher share of non-pooled OOP expenditure in national health financing has a large detrimental effect on adult mortality, controlling for the level of national health expenditure. The implication is that, although incremental health expenditure might improve population health even if such spending is OOP (possibly through additional utilization of healthcare by those who can afford to pay), larger health benefits would be obtained if the extra funds were channelled through pooled prepaid sources. Thus, in addition to their potential financial protection benefits *per se*, pooled prepayment mechanisms seem more likely to maximize the efficiency of additional health funds in terms of generating better population outcomes than extra OOP spending.

Our finding that immunization coverage is linked more robustly to adult health than to child health may be at least partly explained by the fact that broader immunization coverage is frequently achieved through public health campaigns undertaken by governments. Therefore, most of the immediate health benefits of immunization for children may end up being captured by the government health spending variable that is included in our models (i.e. through higher public spending). For people aged 15–60 years, however, the immunization variable seems more likely to act as a proxy for the overall conditions of access to care in the health system, rather than to capture the direct health benefits of vaccination. In our sample, rates of immunization show a strong degree of correlation with other frequently used indicators of care access, including the share of births attended by skilled personnel, physician density and out-patient visits. The robust finding of improved adult health linked to higher immunization coverage therefore confirms the suggestion that broader access to healthcare is an important instrument for countries to achieve better population health.

Some limitations that were imposed on our study by the data that were available must be acknowledged. First, although we use some widely cited health coverage measures, the analysis would have been richer if panel data on other potential coverage proxies—such as measures of out-patient and in-patient visits, catastrophic health spending incidence and barriers to access to healthcare—were available for a reasonable number of countries during the study period. Unfortunately, indicators that are usually employed in cross-sectional country comparisons (e.g. births attended by skilled personnel and out-patient visits at health centres) are not available as a usable time series for most countries. International agencies could play a key role in collating and disseminating the growing amount of information that is provided by national level annual surveys, which often contain questions on issues such as effective access to care (International Household Survey Network: http://www.internationalsurveynetwork.org). Also, for this study we assess population health by using mortality indicators. The analysis would be enriched if a broader set of indicators were available on aspects such as health-related quality of life and equity, which are explicit objectives in many health systems (World Health Organization, 2010a). Although the quality of the data sets that we use can be challenged, they nevertheless represent the most complete time series information available at the country level, and our IV estimation should be an appropriate strategy for dealing with any confounding effects of measurement error in the analyses.

Second, in common with the previous cross-country literature in the area, our research has no overt natural experiment to exploit to identify the effects of changes in health coverage on population outcomes. We contribute to the available literature in the field by using an IV approach that attempts to mimic a natural experiment and is appropriate to our context, explicitly dealing with simultaneity issues and relevant time varying confounders. We also subject the results to a battery of diagnostic, specification and robustness checks. Of course, as in most applied work, this thorough statistical testing cannot entirely rule out, for example, the possibility that covariates such as GDP *per capita* are endogenous themselves, potentially undermining the exogeneity of our two IVs. Further research could explore these matters by seeking to estimate full structural models where each of the variables in question is treated as endogenous, although it is important not to underestimate the challenges of finding plausible variables that could satisfy the necessary exclusion restrictions. In any case, the comparison of our IV estimation results with those from standard fixed effects estimations highlights the need for future studies in the topic to deal explicitly with the potential influence of reverse causal effects, as well as other observable and unobservable confounding factors, to go beyond the identification of mere associations in the data.

Finally, it is worth stressing that our estimates reflect what would happen *on average* to population outcomes if countries experienced variations in health coverage (and if current health programmes were proportionally scaled up). Of course, there are likely to be particular country idiosyncrasies hidden by these averages. The positive effect of incremental publicly pooled expenditure on mortality is probably not an inevitable outcome. Some indicative evidence has suggested that the quality of governance and national institutions (stability of the political system, degree of public sector accountability and so forth) may influence the effectiveness of public spending, and that the beneficial effects of government health expenditures on child and maternal outcomes may be larger in better governed and more equitable countries (Wagstaff and Claeson, 2004; Tolmie, 2007). In our sample, even though the examination of regression residuals shows that our econometric models do a good job in predicting the observed mortality outcomes across countries, there are some countries for which relatively large residuals indicate that there may be other factors influencing observed mortality, beyond those accounted for in our models. As examples among LMICs, Burundi and Malawi consistently exhibit observed under-5 mortality rates that are lower than the levels predicted by the models, whereas the opposite is true for countries like Trinidad and Tobago, Equatorial Guinea and some Middle-Eastern nations. It would be valuable for future studies to examine possible reasons for these outliers, including the influence of specific institutional and governance arrangements, by using case-study methodologies.

World Health Organization (2010) claimed that many countries are still lacking the necessary investment in the health sector that is needed to improve population outcomes in line with the millennium development goals. Our study offers hard evidence that investing in broader health coverage can generate significant gains in terms of population health. Therefore, if such improvements are policy priorities, we believe that countries with sufficient resources should regard enhancements in health system coverage as a key investment target, and the international community should assist the poorest countries in moving towards broader coverage over the coming years.
