# Supplementary material for: Broader health coverage is good for the nation's health: evidence from country level panel data
Source: J R Stat Soc Ser A Stat Soc. 2014 Feb 19;178(1):101–24. doi: 10.1111/rssa.12048 (PMC4280714; doi:10.1111/rssa.12048)
Supplement: Supplementary file 1 [file rssa0178-0101-sd1.pdf]

Appendix 1

Table A1: Variable definitions and sources

Variable	Description	Source
Outcomes		
Under-five mortality rate	Mortality rate, children under five years old (per 1,000 live births)	Institute of Health Metrics and Evaluation
Female mortality rate (adult)	Adult mortality rate, ages 15-60, female (per 1,000 female adults)	World Bank (World Development Indicators)
Male mortality rate (adult)	Adult mortality rate, ages 15-60, male (per 1,000 male adults)	World Bank (World Development Indicators)
Regressors		
Government health spending per capita	Health expenditure per capita: general government (constant 2005 international dollars)	WHO (Global Health Observatory)
VHI health spending per capita	Health expenditure per capita: private pre-paid plans (constant 2005 international dollars)	WHO (Global Health Observatory)
OOP health spending per capita	Health expenditure per capita: private out-of-pocket (constant 2005 international dollars)	WHO (Global Health Observatory)
OOP health spending (share of total)	Private out-of-pocket health expenditure as share of total health expenditure (%)	WHO (Global Health Observatory)
Total health spending per capita	Health expenditure per capita: total (constant 2005 international dollars)	WHO (Global Health Observatory)
Immunization coverage	Median immunization coverage: DTP3, HepB, Hib, polio, BCG and measles (% of children aged 1, or 12-23 months for measles)	WHO, World Bank
GDP per capita	GDP per capita (constant 2005 international dollars)	World Bank (World Development Indicators)
Primary education enrolment rate	Primary school enrolment (% of relevant age group)	World Bank (World Development Indicators)
Population 0-14	Population ages 0-14 (% of total population)	World Bank (World Development Indicators)
Population 65+	Population ages 65 and above (% of total population)	World Bank (World Development Indicators)
CO2 emissions per capita	Carbon dioxide (CO2) emissions (metric tons per capita)	World Bank (World Development Indicators)
Conflict deaths	Battle-related deaths in conflicts	Uppsala Conflict Data Program, Uppsala University

Appendix 2

The causal effect of mortality outcomes on health coverage: full results

This appendix presents and discusses in further detail the results of our IV estimates of the effects of mortality on coverage indicators.

The diagnostic tests presented in Table A2 suggest that CO2 emissions and conflict deaths are relevant to predict mortality outcomes: for all models, the tests reject the joint statistical insignificance of the instruments and first stage model under-identification at conventional levels. Furthermore, Hansen over-identification tests cannot reject the null hypothesis of exogenous instruments by a large margin, providing statistical support to more intuitive arguments concerning the validity of our chosen instruments.

Table A3 shows the baseline first stage results, where CO2 emissions and conflict deaths are used jointly as predictors of each mortality rate (the models also include the income, education and demographic covariates described in the main text, plus time dummies). The first stage regressions indicate that conflict deaths is a statistically significant predictor of all three mortality rates. Particularly, adult mortality is strongly and positively associated with conflict deaths. Although adult mortality rates do not seem significantly influenced by CO2 emissions, the latter instrument is a marginally statistically significant predictor of the under-five mortality rate, with a p-value of 0.108. This suggests that such instrument may be relevant for under-five mortality, and this is reinforced by the result of the Breusch LM test of instrument redundancy (Baum et al. 2007). The null hypothesis of redundancy of the CO2 emissions instrument is rejected at the 8% level of confidence, indicating that the CO2 emissions variable improves the asymptotic efficiency of the estimation.

The reduced form regressions are presented in Panel A of Tables A4, A5 and A6 (for under-five, adult female, and adult male mortality, respectively). These estimations confirm the conclusions from the first stage regressions. While the conflict deaths variable tends to have stronger associations with coverage indicators in the adult mortality models (judged by statistically significant effects or otherwise low p-values from significance tests), CO2 emissions is the stronger instrument in the under-five mortality model for a key coverage measure, namely government health spending. As discussed in the main text, the negative signs of the estimated CO2 coefficients in the under-five mortality regressions (Tables A3 and A4) suggest that CO2 emissions may be capturing the effects of expanded urban agglomerations across countries over the years, favoring reductions in the incidence of waterborne illnesses and diseases associated with vector density, which in turn may allow governments to spend less treating such conditions and transfer those resources to other areas of the public sector.

We present in Tables A4 to A6 the results of additional estimations assessing the robustness of the estimated mortality effects to the presence of a weaker instrument, and further (proxy) testing the validity of our two selected instruments for mortality

rates. In each of these three tables, Panel B shows the results of estimating just-identified versions of equation (4) through IV-2SLS using only the stronger, more relevant excluded instrument for each mortality rate: CO2 emissions for under-five mortality, and conflict deaths for adult mortality rates. Panel C then estimates similar specifications but adds the corresponding weaker instrument (conflict deaths in the case of under-five mortality, and CO2 emissions for adult mortality models) to the right-hand side of equation (4), i.e. as a covariate in X_{it}.

The results of the additional estimations above are remarkably similar to the baseline ones presented in Table A2, with or without the weaker instrument included as covariate. In all but one case the weaker instrument included as covariate is not statistically significant at conventional confidence levels, indicating that there are generally no systematically important effects of these instruments on coverage indicators beyond their effects through mortality. This adds support to the conclusions from the Hansen over-identification tests in Table A2 pointing to the validity of our instruments. These just-identified IV regressions also confirm—and in fact strengthen—the conclusion that under-five mortality has a systematic and positive reverse causal effect on government health expenditures. It is worth noting that, as in Tables A4 to A6, inference regarding the reverse causal effects of mortality on coverage in our baseline models is based on statistical testing robust to the presence of weak instruments (Stock and Wright 2000). Thus, our original conclusions from the IV models in Table A2 using CO2 emissions and conflict deaths jointly as excluded instruments should hold even in the presence of the weaker instrument for each mortality measure.

We also test the robustness of our baseline estimates of coverage effects on mortality (presented in Table 2 in the main text) to changing the instrument set in the first step of our IV approach. That is, we first estimate equation (4) through a just-identified IV-2SLS procedure where each mortality rate is instrumented only by its stronger instrument, as described above (i.e. the models presented in Panel B of Tables A4-A6). We then proceed to the second step of our IV approach using these new estimates to generate instruments as per equation (5), and estimate the effects of coverage on mortality as per equation (3) using these generated instruments. This new set of estimates is presented in Table A7, and compared to the baseline results discussed in the main text. The table shows that, if anything, our baseline IV results of the effects of coverage on mortality rates are further strengthened if we use a just-identified IV model to estimate the reverse causal effects of mortality on coverage. The new point estimates for adult mortality rates are very similar to the baseline values, whereas the point estimate for the effect of government health spending on under-five mortality becomes substantially larger, reflecting the also larger and positive estimate for the reverse causal effect of the under-five mortality rate on government expenditure from Table A4.

As a final specification test, we have sought to estimate alternative IV models using, as additional instruments for mortality rates in equation (4), the number of deaths from road traffic injuries and accidental poisoning. Deaths by such external causes are likely correlated with aggregate mortality rates but, arguably, are unlikely to affect health coverage measures directly, or be themselves influenced by coverage indicators. However, the corresponding information—from WHO International

Classification of Diseases data—is only available for less than half of our original sample (75 countries and 668 observations). Moreover, about 60% of the observations belong to high income countries, with complete data for just 14 low and lower-middle income countries. Thus, we only briefly comment on these results. Unsurprisingly, the number of conflict deaths becomes a weak instrument for under-five and adult mortality in this higher income sub-sample. Only CO2 emissions and accidental poisoning deaths pass most of the under-identification tests. The alternative IV results from the estimation of equation (3) for this selected sub-sample (using CO2 emissions and poisoning deaths as the excluded instruments) tend to agree with our original conclusions as far as pooled health spending is concerned. They reinforce the importance of accounting for the reverse causal effect of under-five mortality on government health expenditures (coefficient = 0.499; p-value = 0.074). Higher government spending is then found to significantly reduce under-five mortality (coefficient = -1.473; p-value = 0.095) and adult male mortality (coefficient = -7.994; p-value = 0.099), with a negative yet statistically insignificant effect on adult female mortality (coefficient = -9.351; p-value = 0.271). The coefficients for the remaining coverage indicators across mortality models are, however, very imprecisely measured. Therefore, compared to our original results, the estimates for this higher income sample tend to support the conclusion that the health gains of incremental coverage measured by pooled health expenditure are larger on the margin for poorer than richer countries.

Table A2: Results for the effects of mortality outcomes on health coverage – 2nd stage

	Government health spending	OOP health spending	VHI health spending	Immunization coverage
	Dependent variable			
	IV-GMM	IV-GMM	IV-GMM	IV-GMM
	(1)	(2)	(3)	(4)
Under-five mortality rate	0.504	0.019	0.044	0.003
	[0.145]	[0.320]	[0.454]	[0.660]
Country fixed effects	Yes	Yes	Yes	Yes
Year fixed effects	Yes	Yes	Yes	Yes
Excluded instruments: first stage F test (statistic)	3.07	3.16	3.16	3.07
Excluded instruments: first stage F test (p-value)	0.049	0.045	0.045	0.049
First stage under-identification $\chi2$ test (statistic)	6.26	6.44	6.44	6.26
First stage under-identification $\chi2$ test (p-value)	0.044	0.040	0.040	0.044
Over-identification Hansen J test (p-value)	0.400	0.328	0.574	0.352
F statistic: second stage	5.75	10.85	1.34	5.39
F statistic: second stage (p-value)	0.000	0.000	0.170	0.000
Number of countries	153	153	153	153
Observations	1,398	1,397	1,397	1,398

	Government health spending	OOP health spending	VHI health spending	Immunization coverage
	IV-GMM	IV-GMM	IV-GMM	IV-GMM
	(1)	(2)	(3)	(4)
Female mortality rate (adult)	0.010	0.007	-0.001	0.008
	[0.588]	[0.301]	[0.329]	[0.820]
Country fixed effects	Yes	Yes	Yes	Yes
Year fixed effects	Yes	Yes	Yes	Yes
Excluded instruments: first stage F test (statistic)	7.34	7.19	7.19	7.34
Excluded instruments: first stage F test (p-value)	0.001	0.001	0.001	0.001
First stage under-identification $\chi2$ test (statistic)	15.00	14.70	14.70	15.00
First stage under-identification $\chi2$ test (p-value)	0.001	0.001	0.001	0.001
Over-identification Hansen J test (p-value)	0.598	0.281	0.237	0.889
F statistic: second stage	26.84	8.62	1.93	5.35
F statistic: second stage (p-value)	0.000	0.000	0.017	0.000
Number of countries	148	148	148	148
Observations	1,223	1,222	1,222	1,223

	Government health spending	OOP health spending	VHI health spending	Immunization coverage
	IV-GMM	IV-GMM	IV-GMM	IV-GMM
	(1)	(2)	(3)	(4)
Male mortality rate (adult)	0.009	0.005	-0.004	0.008
	[0.588]	[0.301]	[0.329]	[0.820]
Country fixed effects	Yes	Yes	Yes	Yes
Year fixed effects	Yes	Yes	Yes	Yes
Excluded instruments: first stage F test (statistic)	12.02	11.60	11.60	12.02
Excluded instruments: first stage F test (p-value)	0.000	0.000	0.000	0.000
First stage under-identification $\chi2$ test (statistic)	24.57	23.71	23.71	24.57
First stage under-identification $\chi2$ test (p-value)	0.000	0.000	0.000	0.000
Over-identification Hansen J test (p-value)	0.553	0.210	0.273	0.884
F statistic: second stage	25.48	9.23	1.21	5.16
F statistic: second stage (p-value)	0.000	0.000	0.263	0.000
Number of countries	148	148	148	148
Observations	1,223	1,222	1,222	1,223

Notes: Time period is 1995-2008. Models estimated by instrumental variables through a two-step generalized method-of-moments approach (IV-GMM), using as instruments CO2 emissions per capita and the number of battle-related deaths in internal or international conflicts. All regressions also control for GDP per capita, the primary education enrolment rate, the share of population aged 0-14, and the share of population aged over 65. P-values (in square brackets under coefficients) are from two-sided *t* tests with standard errors robust to the presence of weak instruments (Stock and Wright 2000) and arbitrary heteroskedasticity and autocorrelation.

Table A3: Results for the effects of mortality outcomes on health coverage – 1st stage

	Under-five mortality rate	Female mortality rate (adult)	Male mortality rate (adult)
	FE-LS	FE-LS	FE-LS
	(1)	(2)	(3)
CO2 emissions	-0.269	-0.133	0.458
	(0.108)	(0.737)	(0.328)
Conflict deaths	**-0.002**	**0.034**	**0.032**
	**(0.035)**	**(0.000)**	**(0.000)**
Country fixed effects	Yes	Yes	Yes
Year fixed effects	Yes	Yes	Yes
Joint Wald significance test of two variables (*F* statistic)	3.16	7.19	11.60
Joint Wald significance test of two variables (p-value)	0.045	0.001	0.000
LM test of redundancy of CO2 emissions instrument (p-value)	0.079	0.733	0.337
Number of countries	153	148	148
Observations	1,397	1,222	1,222

Notes: Time period is 1995-2008. Models estimated by standard least squares fixed effects (FE-LS), controlling also for GDP per capita, the primary education enrolment rate, the share of population aged 0-14, and the share of population aged over 65. P-values (in parentheses under coefficients) are from two-sided *t* tests with standard errors robust to arbitrary heteroskedasticity and autocorrelation. Bold entries indicate coefficients statistically significant at the 10% level of confidence or below.

Table A4: Further results for the effects of mortality outcomes on health coverage – under-five mortality

	Dependent variable											
	Panel A: Reduced form				Panel B: Just-identified model with stronger instrument				Panel C: Just-identified model with stronger instrument (weaker instrument included as covariate)			
	Government health spending	OOP health spending	VHI health spending	Immunization coverage	Government health spending	OOP health spending	VHI health spending	Immunization coverage	Government health spending	OOP health spending	VHI health spending	Immunization coverage
	FE-LS	FE-LS	FE-LS	FE-LS	IV-2SLS	IV-2SLS	IV-2SLS	IV-2SLS	IV-2SLS	IV-2SLS	IV-2SLS	IV-2SLS
	(1)	(2)	(3)	(4)	(5)	(6)	(7)	(8)	(9)	(10)	(11)	(12)
Under-five mortality rate					**0.700**	0.052	0.073	0.009	**0.700**	0.052	0.073	0.009
					**[0.055]**	[0.364]	[0.279]	[0.847]	**[0.055]**	[0.370]	[0.279]	[0.840]
CO2 emissions	**-0.188**	-0.014	-0.020	-0.002								
	**(0.048)**	(0.358)	(0.317)	(0.842)								
Conflict deaths	0.0001	0.0001	-0.0001	**0.0011**					0.0018	0.0002	0.0001	**0.0011**
	(0.655)	(0.107)	(0.595)	**(0.000)**					(0.213)	(0.178)	(0.475)	**(0.000)**
Country fixed effects	Yes	Yes	Yes	Yes	Yes	Yes	Yes	Yes	Yes	Yes	Yes	Yes
Year fixed effects	Yes	Yes	Yes	Yes	Yes	Yes	Yes	Yes	Yes	Yes	Yes	Yes
Single instrument (stronger)					CO2 emissions	CO2 emissions	CO2 emissions	CO2 emissions	CO2 emissions	CO2 emissions	CO2 emissions	CO2 emissions
First stage under-identification LM test (p-value)					0.078	0.078	0.078	0.078	0.079	0.079	0.079	0.079
Number of countries	153	153	153	153	153	153	153	153	153	153	153	153
Observations	1,398	1,397	1,397	1,398	1,398	1,397	1,397	1,398	1,398	1,397	1,397	1,398

Notes: Time period is 1995-2008. Models in Panel A estimated by standard least squares fixed effects (FE-LS). Models in Panels B and C estimated by instrumental variables through a two-stage least squares approach (IV-2SLS). All regressions control also for GDP per capita, the primary education enrolment rate, the share of population aged 0-14, and the share of population aged over 65. P-values in parentheses under coefficients are from two-sided *t* tests with standard errors robust to arbitrary heteroskedasticity and autocorrelation. P-values in square brackets under coefficients are from two-sided *t* tests with standard errors robust to the presence of weak instruments (Stock and Wright 2000) and arbitrary heteroskedasticity and autocorrelation. Bold entries indicate coefficients statistically significant at the 10% level of confidence or below.

Table A5: Further results for the effects of mortality outcomes on health coverage – adult female mortality

	Dependent variable											
	Panel A: Reduced form				Panel B: Just-identified model with stronger instrument				Panel C: Just-identified model with stronger instrument (weaker instrument included as covariate)			
	Government health spending	OOP health spending	VHI health spending	Immunization coverage	Government health spending	OOP health spending	VHI health spending	Immunization coverage	Government health spending	OOP health spending	VHI health spending	Immunization coverage
	FE-LS	FE-LS	FE-LS	FE-LS	IV-2SLS	IV-2SLS	IV-2SLS	IV-2SLS	IV-2SLS	IV-2SLS	IV-2SLS	IV-2SLS
	(1)	(2)	(3)	(4)	(5)	(6)	(7)	(8)	(9)	(10)	(11)	(12)
Female mortality rate (adult)					0.011	0.007	-0.001	0.008	0.011	0.007	-0.001	0.008
					[0.318]	[0.253]	[0.543]	[0.530]	[0.312]	[0.253]	[0.425]	[0.530]
CO2 emissions	-0.075	-0.020	-0.023	0.001					-0.073	-0.019	-0.023	0.002
	(0.581)	(0.226)	(0.247)	(0.934)					(0.592)	(0.234)	(0.242)	(0.891)
Conflict deaths	0.0004	**0.0002**	-0.0001	0.0003								
	(0.129)	**(0.002)**	(0.584)	(0.219)								
Country fixed effects	Yes	Yes	Yes	Yes	Yes	Yes	Yes	Yes	Yes	Yes	Yes	Yes
Year fixed effects	Yes	Yes	Yes	Yes	Yes	Yes	Yes	Yes	Yes	Yes	Yes	Yes
Single instrument (stronger)					Conflict deaths	Conflict deaths	Conflict deaths	Conflict deaths	Conflict deaths	Conflict deaths	Conflict deaths	Conflict deaths
First stage under-identification LM test (p-value)					0.363	0.365	0.365	0.363	0.363	0.365	0.365	0.363
Number of countries	148	148	148	148	148	148	148	148	148	148	148	148
Observations	1,223	1,222	1,222	1,223	1,223	1,222	1,222	1,223	1,223	1,222	1,222	1,223

Notes: Time period is 1995-2008. Models in Panel A estimated by standard least squares fixed effects (FE-LS). Models in Panels B and C estimated by instrumental variables through a two-stage least squares approach (IV-2SLS). All regressions control also for GDP per capita, the primary education enrolment rate, the share of population aged 0-14, and the share of population aged over 65. P-values in parentheses under coefficients are from two-sided *t* tests with standard errors robust to arbitrary heteroskedasticity and autocorrelation. P-values in square brackets under coefficients are from two-sided *t* tests with standard errors robust to the presence of weak instruments (Stock and Wright 2000) and arbitrary heteroskedasticity and autocorrelation. Bold entries indicate coefficients statistically significant at the 10% level of confidence or below.

Table A6: Further results for the effects of mortality outcomes on health coverage – adult male mortality

	Dependent variable											
	Panel A: Reduced form				Panel B: Just-identified model with stronger instrument				Panel C: Just-identified model with stronger instrument (weaker instrument included as covariate)			
	Government health spending	OOP health spending	VHI health spending	Immunization coverage	Government health spending	OOP health spending	VHI health spending	Immunization coverage	Government health spending	OOP health spending	VHI health spending	Immunization coverage
	FE-LS	FE-LS	FE-LS	FE-LS	IV-2SLS	IV-2SLS	IV-2SLS	IV-2SLS	IV-2SLS	IV-2SLS	IV-2SLS	IV-2SLS
	(1)	(2)	(3)	(4)	(5)	(6)	(7)	(8)	(9)	(10)	(11)	(12)
Male mortality rate (adult)					0.012	0.007	-0.001	0.008	0.011	0.007	-0.001	0.008
					[0.318]	[0.253]	[0.543]	[0.530]	[0.312]	[0.253]	[0.425]	[0.530]
CO_2 emissions	-0.075	-0.020	-0.023	0.001					-0.080	-0.023	-0.023	-0.003
	(0.581)	(0.226)	(0.247)	(0.934)					(0.565)	(0.168)	(0.262)	(0.887)
Conflict deaths	0.0004	**0.0002**	-0.0001	0.0003								
	(0.129)	**(0.002)**	(0.584)	(0.219)								
Country fixed effects	Yes	Yes	Yes	Yes	Yes	Yes	Yes	Yes	Yes	Yes	Yes	Yes
Year fixed effects	Yes	Yes	Yes	Yes	Yes	Yes	Yes	Yes	Yes	Yes	Yes	Yes
Single instrument (stronger)					Conflict deaths	Conflict deaths	Conflict deaths	Conflict deaths	Conflict deaths	Conflict deaths	Conflict deaths	Conflict deaths
First stage under-identification LM test (p-value)					0.344	0.348	0.348	0.344	0.344	0.348	0.348	0.344
Number of countries	148	148	148	148	148	148	148	148	148	148	148	148
Observations	1,223	1,222	1,222	1,223	1,223	1,222	1,222	1,223	1,223	1,222	1,222	1,223

Notes: Time period is 1995-2008. Models in Panel A estimated by standard least squares fixed effects (FE-LS). Models in Panels B and C estimated by instrumental variables through a two-stage least squares approach (IV-2SLS). All regressions control also for GDP per capita, the primary education enrolment rate, the share of population aged 0-14, and the share of population aged over 65. P-values in parentheses under coefficients are from two-sided *t* tests with standard errors robust to arbitrary heteroskedasticity and autocorrelation. P-values in square brackets under coefficients are from two-sided *t* tests with standard errors robust to the presence of weak instruments (Stock and Wright 2000) and arbitrary heteroskedasticity and autocorrelation. Bold entries indicate coefficients statistically significant at the 10% level of confidence or below.

Table A7: Robustness of baseline effects of health coverage on mortality to changes in instrument set

	Under-five mortality rate	Female mortality rate (adult)	Male mortality rate (adult)
	IV-2SLS	IV-2SLS	IV-2SLS
	(1)	(2)	(3)
Government health spending			
Baseline	-13.193	-2.583	-2.210
	(0.018)	(0.050)	(0.025)
Using only stronger instrument to estimate equation (4)	-23.298	-2.996	-2.485
	(0.058)	(0.042)	(0.032)
OOP health spending			
Baseline		-23.385	-15.545
		(0.040)	(0.016)
Using only stronger instrument to estimate equation (4)		-23.850	-20.636
		(0.040)	(0.013)
Immunization coverage			
Baseline	-2.203	-9.841	-7.858
	(0.073)	(0.030)	(0.020)
Using only stronger instrument to estimate equation (4)	-2.634	-9.903	-7.687
	(0.191)	(0.030)	(0.022)

Notes: For each health coverage indicator, the first row shows the statistically significant two-stage least squares (IV-2SLS) coefficients found in the baseline models (presented in Table 2), followed by the corresponding coefficients estimated in the robustness test. The stronger instruments are CO2 emissions for under-five mortality, and conflict deaths for adult mortality rates (see text). All regressions also control for GDP per capita, the primary education enrolment rate, the share of population aged 0-14, the share of population aged over 65, and country and year fixed effects. P-values (in parentheses under coefficients) are from two-sided t tests with standard errors robust to arbitrary heteroskedasticity and autocorrelation.

Appendix 3

Economic magnitude of government health spending effects on under-five mortality

This appendix presents details of the back-of-the-envelope calculations of life years saved based on the estimated baseline coefficients for government health spending and under-five mortality in the full sample (Table 2, column 2), and the low and middle income (LMIC) sub-sample (Table 4, column 1). The calculations below are made for a marginal increase of one dollar of government health spending per capita, which is equivalent to relatively small per capita spending increases of 0.2% in the average country in the sample, and 0.7% in the average low and middle income country (or total public health expenditure increases of around $32.5 million and $34.8 million, respectively). We note that the illustrative exercise in this section should be interpreted with due caution and does not intend to serve as a full computation of welfare gains, as it does not compare the costs and benefits of public investments in health vis-à-vis other sectors (such as education, for example).

According to the coefficients from the baseline IV specification for the full sample, and keeping other factors constant, the average country would experience a marginal reduction of 0.132 under-five deaths per 1,000 for an extra dollar of government health spending per capita. We make the conservative assumption that all child deaths occur at age five. From WHO life tables, five-year olds are expected to live an extra 67.5 years (World Health Organization 2010, world figures). Using the average observed under-five mortality rate in the sample, and our estimates of deaths averted among the population aged 0-4, this results in 451 lives saved in a given country. Combined with the expected extra years of life this leads to an estimated 451 x 67.5 = 30,443 life years saved in total. The data on under-five population by country refer to year 2008 and come from UNICEF (2011).

As above, the calculations for the LMIC sub-sample are based on the assumption that all child deaths occur at age five. WHO life tables have separate information on life expectancy at age five for low, lower-middle, upper-middle and high income countries (World Health Organization 2010); we use an average of the life expectancy figures for the first three income categories, weighted by the average under-five population in the sample for each income group. From these calculations, children aged 0-4 in low and middle income countries are expected to live an extra 64.8 years. Combined with the average under-five mortality rate in that group of countries, the relevant estimate of averted under-five deaths (a reduction of 0.908 deaths per 1,000 for an extra dollar of public health spending per capita) results then in 3,707 lives saved in the average low and middle income country, and 240,061 extra life years saved.

Dividing the total extra government health spending by the relevant figures for lives and life years saved, the results suggest public health spending figures per life saved of around $72,000 for the typical country, and $9,400 per life saved for the typical lower income country. Similarly, the marginal cost of saving a year of life amounts

then on average to around $1,000 in the whole sample of countries, and $145 for a low or middle income country.

References

Baum, C., Schaffer, M., and Stillman, S. (2007) Enhanced routines for instrumental variables/GMM estimation and testing. *Stata Journal*, 7(4), 465-506.

Stock, J., and Wright, J. (2000) GMM with weak identification. *Econometrica*, 68(5), 1055-1096.

World Health Organization (2010) *Life tables for WHO member states, 1990, 2000, 2010 – by country and region*. Geneva: World Health Organization.

UNICEF (2011) *Statistics and monitoring database*. New York: UNICEF. (Available from http://www.unicef.org/statistics/index.)
